# HPV-positive status associated with inflamed immune microenvironment and improved response to anti-PD-1 therapy in head and neck squamous cell carcinoma

**DOI:** 10.1038/s41598-019-49771-0

**Published:** 2019-09-16

**Authors:** Jian Wang, Hao Sun, Qin Zeng, Xue-Jun Guo, Hui Wang, Huan-Huan Liu, Zhong-Yi Dong

**Affiliations:** 1 Department of Radiation Oncology, Nanfang Hospital, Southern Medical University, Guangzhou, China; 2 School of Medicine, South China University of Technology, Guangzhou, China

**Keywords:** Predictive markers, Cancer microenvironment

## Abstract

Chemotherapy and radiotherapy predominantly improve the clinical outcomes of patients with human papillomavirus (HPV)-related head and neck squamous cell carcinoma (HNSCC). Whether this superiority goes on when treated with immune checkpoint inhibitors is still unclear. This study sought to determine the predictive value and potential mechanisms of HPV status for the treatment of programmed cell death 1 (PD-1)/ligand 1(PD-L1) inhibitors. We conducted an integrated analysis of the relationships between HPV status and PD-L1, tumor mutation burden (TMB) and inflammation-related immune cells and molecules, based on the analysis of repository databases and resected HNSCC specimens. The pooled analysis of overall survival (OS) and objective response rate (ORR) suggested that HPV-positive patients benefited more from PD-1/PD-L1 inhibitors than HPV-negative patients (OS: hazard ratio (HR) = 0.71, *p* = 0.02; ORR: 21.9% vs 14.1%, odds ratio (OR) = 1.79, *p* = 0.01). Analysis of public databases and resected HNSCC specimens revealed that HPV status was independent of PD-L1 expression and TMB in HNSCC. However, HPV infection significantly increased T-cell infiltration, immune effector cell activation and the diversity of T-cell receptors. Notably, HPV-positivity correlated with increased immune cytolytic activity and a T-cell-inflamed gene expression profile. This work provides evidence that HPV status can be used to predict the effectiveness of PD-1 inhibitors in HNSCC, independently of PD-L1 expression and TMB, and probably results from an inflamed immune microenvironment induced by HPV infection.

## Introduction

Head and neck squamous cell carcinoma (HNSCC) includes cancers of the oral cavity, oropharynx, and larynx, and accounts for approximately 4% of cancers worldwide^1^. Because the majority of HNSCC patients are diagnosed at an advanced stage (stage III to IVB), HNSCC is associated with poor prognosis and a high mortality rate. Traditional treatment strategies typically consist of doublet platinum-based chemotherapy, which is associated with significant toxicity and has limited therapeutic effect^2^. Novel therapeutic strategies that can broaden the current treatment schedule of HNSCC are urgently needed.

The development of immune checkpoint inhibitors (ICIs) has revolutionized immunotherapy. Anti- programmed cell death 1 (PD-1)/ligand 1(PD-L1) therapy is effective in multiple tumor types, and can bring remarkable clinical benefits with limited toxicity. Several clinical trials have proved that anti-PD-1/PD-L1 therapy results in clinically meaningful antitumor activity and an acceptable safety profile when used to treat HNSCC patients^3–5^. However, the response rate to PD-1/PD-L1 blockade among biomarker-unselected groups is still far from satisfactory. Nowadays, PD-L1 levels are usually determined by immunohistochemical (IHC) staining in clinical practice, but prediction of the response to anti-PD-1/PD-L1 is imperfect owing to dynamic and heterogeneous expression^6^. Because the immune environment of HNSCC is quite complicated, a more comprehensive understanding is needed to guide new ICI applications.

Human papillomavirus (HPV)-associated HNSCC appears to have distinct biological and clinical features; it is associated with better prognosis than HPV-negative HNSCC^7,8^. The primary clinical data have revealed that the response rate to PD-1/PD-L1 is higher in HPV-positive patients than in HPV-negative patients^3,4^. HPV status may be another factor that can be used to classify HNSCC and identify patient suitability. However, the underlying mechanism and the potential association between HPV status and the tumor immune environment have not yet been fully characterized.

In this study, we attempted to elucidate the relationship between HPV status and immune environment elements that account for the response to PD-1/PD-L1 blocking. We conducted an integrated analysis incorporating HPV status, PD-L1 expression, content of CD8 + T-cell infiltration and tumor mutation burden (TMB), based on data from a multicenter database. Finally, we determined that an HPV-positive status contributes to T-cell infiltration and enhances cytolytic activity (CYT), which results in a better response to anti-PD-1/PD-L1 therapy in HNSCC patients.

## Patients and Methods

### Clinical cohorts

Data for The Cancer Genome Atlas (TCGA), GSE40774 and Memorial Sloan Kettering-Integrated Mutation Profiling of Actionable Cancer Targets (MSK-IMPACT)^9^ cohorts were retrieved from online data repositories. The TCGA cohort comprises 451 HNSCC patients, 155 with confirmed HPV status. HPV positive was defined as a positive p16 IHC or HPV *In-Situ* Hybridization (ISH) result or an HPV viral titer over 30. We retrieved their RNA and protein expression profiles, copy number alteration (CNA) information and gene mutation data from the cBioPortal website. The GSE40774 cohort comprises 134 HNSCC patients, and we obtained their associated data from the Gene Expression Omnibus (GEO) database, including detailed information about each patient’s HPV status and RNA sequencing. A total of 52 HNSCC patients had confirmed HPV status, and the associated CNA information and gene mutation profiles were extracted from the MSK-IMPACT cohort as a subgroup. HNSCC-tissue microarray (TMA) cohorts containing a total of 130 tissues were obtained from Shanghai Biochip Co., Ltd (Horac080PG01) and Alenabio Biotechnology Co., Ltd (Xian, China; HN601b). All patients provided specimens for HNSCC-TMA with written informed consent. Human tumor samples from TCGA and GEO database were available of patient consent and tumor quality. Additional publicly available data sets used in this study are listed in Supplementary Table S1. The key variables of these four cohorts, including demographic and clinical data, are provided in Supplementary Table S2.

### Pooled analysis

We carried out a pooled analysis of the efficacy of PD-1/PD-L1 inhibitors in HPV-positive and -negative HNSCC patients. We analyzed the OS data for 425 patients from four trials (CheckMate-141^4^, KEYNOTE-012^3^, KEYNOTE-055^5^, and NCT01693562 (HAWK)^10^) and the ORR data for 589 patients from six trials (CheckMate-141, KEYNOTE-012, KEYNOTE-012 Expansion^11^, KEYNOTE-055, NCT01693562, and NCT01375842^12^). The baseline characteristics of the enrolled trials are summarized in Supplementary Table S3.

Data extraction from eligible studies was performed independently by two authors (Xue-Jun Guo and Qin Zeng). Hazard ratios for the OS analysis were calculated using the Tierney methodology if not immediately available from the primary report^13^.

### Immunohistochemistry

Samples for HNSCC-TMA were collected using 1.5-mm diameter core needles from tumor regions with the most representative histology of each surgical specimen. Serial sections from the HNSCC-TMA were used for analyzing PD-L1, p16 (HPV) and CD8. Tumor sections were assessed immunohistochemically using PD-L1 (clone: SP142, Spring Bioscience, Inc.), CD8 (clone: C8/144B, Gene Tech Co., Ltd.) and p16^Ink4a^ antibodies (clone G175-405, BD Biosciences PharMingen, San Diego, CA, USA). The IHC-stained tissue sections were scored separately by two pathologists blinded to the clinical parameters. The PD-L1 expression of tumor cells and immune cells was evaluated using a three-tiered grading system: tumor cell (TC) 3/immune cell (IC) 3: ≥50% for TC or ≥10% for IC; TC2/IC2: 5–49% for TC or 5–9% for IC; TC0-1/IC0-1: <5% for TC or IC. We assessed the percentage of CD8+ lymphocytes among all nucleated cells in the stromal compartments. Scoring cut-off points were set at 10% or 25% for each core, according to the cell density: low density: <10%; moderate density: 10–25%; high density: ≥25%^14,15^. Positive p16 expression was defined as strong and diffuse nuclear and cytoplasmic staining in ≥70% tumor cells^16^. The patients and experiments included in this study were approved by the Institutional Ethical Board (IRB) of Nanfang Hospital. We confirmed that all experiments were performed in accordance with relevant guidelines and regulations.

### Mutation burden, copy number alteration (CNA) and neoantigen analysis

The somatic mutation and CNA data for HNSCC patients in the TCGA cohort were retrieved from the TCGA database portal (https://tcga-data.nci.nih.gov/tcga/findArchives.htm). The mutation and CNA data for the MSK-IMPACT cohort were retrieved from the cBioPortal for Cancer Genomics (http://www.cbioportal.org/study?id=msk_impact_2017#summary). To assess the mutation burden, the number of mutated genes carrying at least one non-synonymous mutation in the coding region was computed for each tumor. Tumor neoantigens of HNSCC patients in the TCGA cohort were directly obtained from the supplementary materials provided in a previous published study^17^. If the mutation was predicted to produce a “binder” neopeptide with affinity <500 nM and its corresponding gene expression was greater than 10 Transcripts Per Million (TPM), the mutation would be designated putatively antigenic.

### RNA expression profiling analysis

The gene expression data for TCGA cohort and GEO cohorts (GSE40774 and GSE62027) were downloaded from TCGA database portal and GEO repository (https://www.ncbi.nlm.nih.gov/geo) respectively. Cytolytic activity (CYT) was defined as the log averages (geometric means) of *GZMA* and *PRF1* RNA expression data in terms of TPM as the previous study suggested^17^. The T cell-inflamed gene expression profile (GEP) was composed of 18 genes, including *CCL5*, *CD27*, *CD274*, *CD276*, *CD8A*, *CMKLR1*, *CXCL9*, *CXCR6*, *HLA-DQA1*, *HLA-DRB1*, *HLA-E*, *IDO1*, *LAG3*, *NKG7*, *PDCD1LG2*, *PSMB10*, *STAT1*, and *TIGIT*^18^. For the calculation of GEP score in TCGA and GSE40774, we used Reads per kilobase of exon per million reads mapped (RPKM) log2 intensity data for each gene, and then averaged the expression for genes in the signature geneset to obtain a signature score per patient.

### T Cell Receptor (TCR) Analysis

Data of TCR diversity and richness of HNSCC was directly obtained from previous published article^19^, which was analyzed from the TCGA RNA-Seq dataset. Briefly, identification of TCR CDR3 sequences from T cells present in the sequenced tumor sections was performed using MiTCR v1.0.3^20^. Paired-end fastq files were concatenated into a single file and run through MiTCR using the appropriate parameter set for the sequence read length as described in Brown *et al*. Runs were performed on the ISB Cancer Genomics Cloud.

### Statistical analyses

Statistical analyses were performed using Review Manager Version 5.2 (RevMan, Cochrane Collaboration), GraphPad Prism (version 7.01) and SPSS version 22.0 (SPSS, Inc.). Statistical heterogeneity in the pooled analysis was evaluated using the chi-squared (χ^2^) test and inconsistency index (*I*^2^); values were considered significant when the χ^2^ P-value was <0.1 or when *I*^2^ was >50%. In the absence of statistically significant heterogeneity, the fixed-effect model was used for pooled analysis. Otherwise, the random-effect model was selected. Chi-squared tests were used to analyze differences in the expression levels of PD-L1 and CD8 between the HPV-positive and -negative groups. The correlation between HPV viral titers and immune-related parameters was analyzed by Spearman’s rank correlation. All reported P-values were two-tailed, and for all analyses P ≤ 0.05 was considered statistically significant, unless otherwise specified.

## Results

### Patients with HPV-positive HNSCC showed favorable response to PD-1/PD-L1 inhibitors

**Figure 1:**
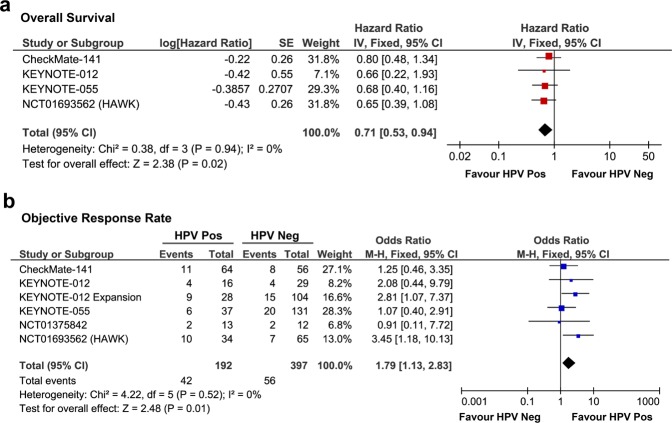
Forest plots of hazard ratios (HRs) for overall survival (**a**) and odds ratios (ORs) for objective response rate (**b**) from six clinical trials, comparing human papillomavirus (HPV)-positive with HPV-negative head and neck squamous cell carcinoma (HNSCC) patients treated with programmed cell death 1 (PD-1)/ligand 1(PD-L1) inhibitors. Pooled HRs and ORs were computed using the fixed-effects model. Pos, positive; Neg, negative.

It has been proven that an HPV-positive status is associated with better clinical outcomes for HNSCC patients undergoing chemotherapy or radiotherapy. Therefore, we hypothesized that HPV status could also be used as a biomarker for anti-PD-1/PD-L1 therapy. We conducted a pooled analysis to assess the efficacy of a PD-1/PD-L1 inhibitor in HPV-positive and -negative HNSCC patients. A total of 425 patients who received treatment with PD-1/PD-L1 inhibitors and had confirmed HPV status from four clinical trials were included in the analysis. The pooled analysis revealed that HPV-positive patients experienced greater clinical benefits in terms of overall survival (OS) than HPV-negative patients (hazard ratio (HR) = 0.71; 95% confidence interval (95%CI) = 0.53–0.94; *p* = 0.02) (Fig. 1a). Further analysis of 589 patients from six trials revealed a higher objective response rate (ORR) in HPV-positive HNSCC patients than in HPV-negative patients (ORR: 21.9% vs 14.1%, odds ratio (OR) = 1.79, 95%CI = 1.13–2.83; *p* = 0.01) (Fig. 1b**)**. These results indicate that HPV-positive status may be a potential biomarker for the application of anti-PD-1/PD-L1 therapy, and HPV-related HNSCC patients could benefit from PD-1 blockade.

### HPV as a predictive biomarker was independent of PD-L1 expression

**Figure 2:**
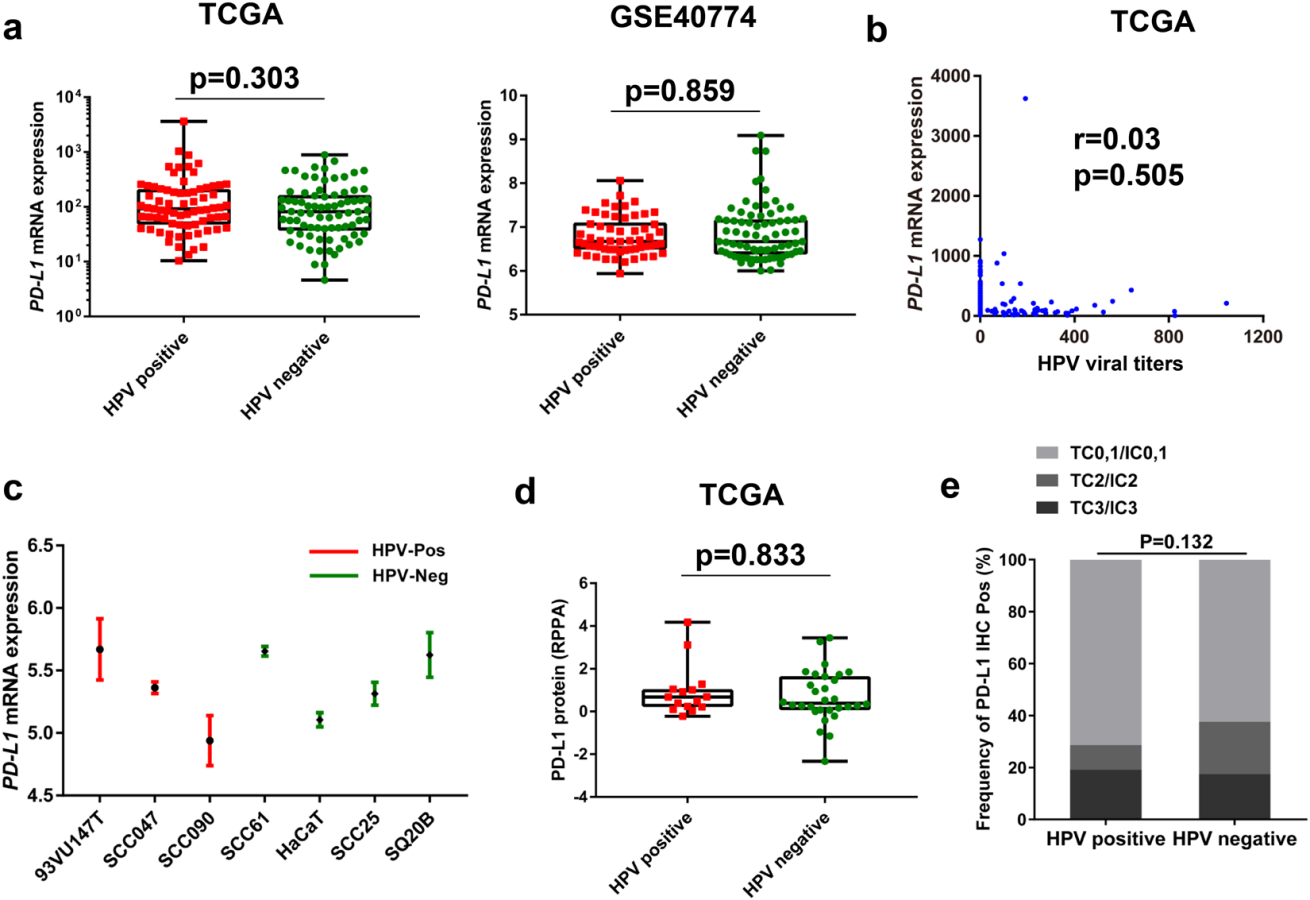
HPV-positive status was independent of PD-L1 expression in patients with head and neck squamous cell carcinoma (HNSCC). (**a**) Quantitative analysis of *PD-L1* RNA expression from RNA-seq profiles in patients with HPV-positive and -negative HNSCC based on The Cancer Genome Atlas (TCGA) and Gene Expression Omnibus (GEO: GSE40774) database. (**b**) Correlation analysis of HPV viral titers and PD-L1 expression based on the TCGA cohort. (**c**) PD-L1 levels were analyzed in seven HNSCC cell lines with confirmed HPV status based on GSE62027 RNA-seq profiles. (**d**) Quantitative analysis of PD-L1 protein expression derived from reverse phase protein array according to HPV status. (**e**) Immunohistochemical (IHC) analysis of PD-L1 protein expression based on HPV status in a cohort of 130 resected HNSCC patients. TC, tumor cell; IC, immune cell; Pos, positive; Neg, negative.

Previous studies have demonstrated that the expression of PD-L1 is a useful predictive biomarker for the effectiveness of checkpoint inhibitors in many cancer types^21–23^. We speculated whether the use of HPV status as a predictive biomarker is dependent on PD-L1 expression. To verify this hypothesis, we conducted an analysis based on gene profiles from The Cancer Genome Atlas (TCGA) and GSE40774 cohorts to compare the RNA profiles of PD-L1 in HPV-positive and -negative HNSCC patients. The results revealed that there was no significant difference in PD-L1 expression between the HPV-positive and -negative groups (TCGA: *p* = 0.303; GSE40774: *p* = 0.859; Fig. 2a**)**. A correlation study of HPV viral titers and PD-L1 expression in the TCGA cohort also revealed poor correlation (r = 0.03, *p* = 0.505; Fig. 2b**)**. We further analyzed PD-L1 levels among seven HNSCC cell lines based on the RNA sequencing profiles of the GSE62027 cohort (three HPV-positive: 93VU147T, SCC047, SCC090; four HPV-negative: SCC61, HaCaT, SCC25, SQ20B) and found no distinct relationship between HPV status and PD-L1 expression in the HNSCC cell lines (Fig. 2c).

A reverse-phase protein array (RPPA) analysis of 207 HNSCC patients from the TCGA cohort revealed no significant correlation between HPV status and PD-L1 protein expression (*p* = 0.833; Fig. 2d). Furthermore, IHC analysis were also carried out to reveal PD-L1 and p16 protein expression in a cohort comprising 130 resected HNSCC specimens. Consistent with these results, there was also no difference in PD-L1 immunostaining between the HPV-positive HNSCC and HPV-negative HNSCC groups (*p* = 0.132; Fig. 2e). The results described above indicate that HPV status as a potential predictive biomarker for anti-PD-1/PD-L1 therapy in HNSCC patients is independent of PD-L1 expression.

### HPV infection did not directly increase the tumor mutation burden (TMB) of HNSCC patients

**Figure 3:**
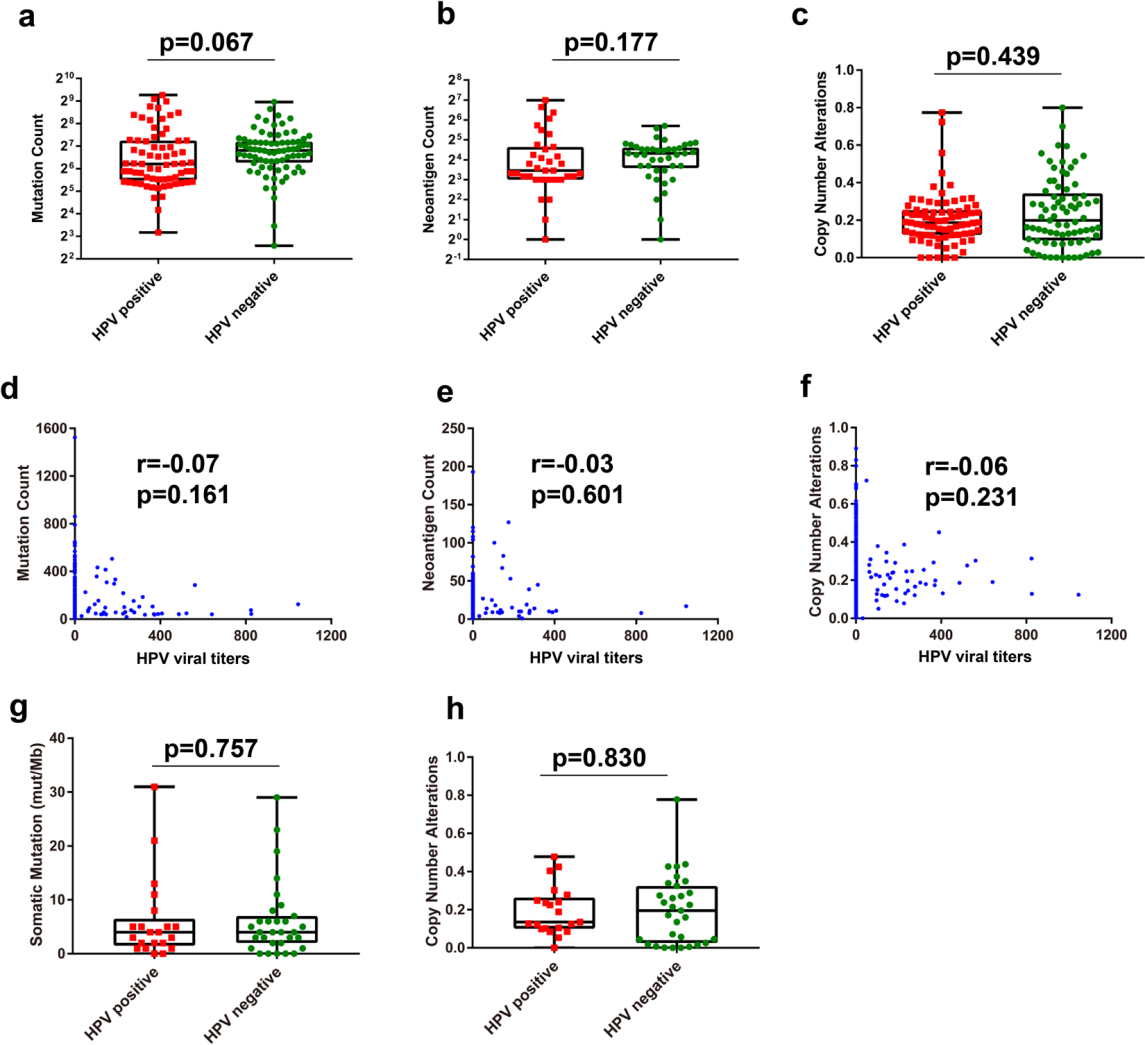
HPV infection did not directly impact tumor mutation profiles of head and neck squamous cell carcinoma (HNSCC) patients. (**a**–**c**) Quantitative analyses of tumor mutational burden (TMB), neoantigens count, and copy number alteration (CNA) in HPV-positive and -negative tumors based on the TCGA cohort. (**d**–**f**) Correlation analyses of HPV viral titers and TMB, neoantigen count, and CNA based on the TCGA cohort. (**g**,**h**) Quantitative analyses of TMB and CNA in HPV-positive and -negative HNSCC based on the MSK-IMPACT cohort.

Previous studies have demonstrated that viral infections in HNSCC, such as Epstein-Barr virus (EBV) and HPV, were associated with chromosomal instability and DNA damage repair^24–26^, which may cause increased TMB. In this study, we aimed to determine whether HPV infection elevates the TMB of HNSCC patients. We analyzed the relationships between HPV status, mutation profiles and CNA in HNSCC patients with the TCGA and MSK-IMPACT cohorts. Analysis data obtained from the TCGA cohort showed that HPV did not increase the total mutation count (*p* = 0.067; Fig. 3a), tumor neoantigens (*p* = 0.117; Fig. 3b) or CNA (*p* = 0.439; Fig. 3c) compared with the HPV-negative subgroup. We also investigated the correlation between HPV viral titers and tumor genomic alterations mentioned above, and found no correlation between HPV viral titers and total mutation count (r = 0.07, *p* = 0.161; Fig. 3d), tumor neoantigens (r = 0.03, *p* = 0.601; Fig. 3e) or CNA (r = 0.06, *p* = 0.231; Fig. 3f). Consistent with the results obtained from the TCGA cohort, there were no significant differences in total mutation count (*p* = 0.757; Fig. 3g) or CNA (*p* = 0.830; Fig. 3h) between the HPV status groups in the MSK-IMPACT cohort. These findings suggest that HPV infection does not directly increase the TMB or neoantigen count of HNSCC patients.

### HPV infection promoted T-cell infiltration and immune recognition

**Figure 4:**
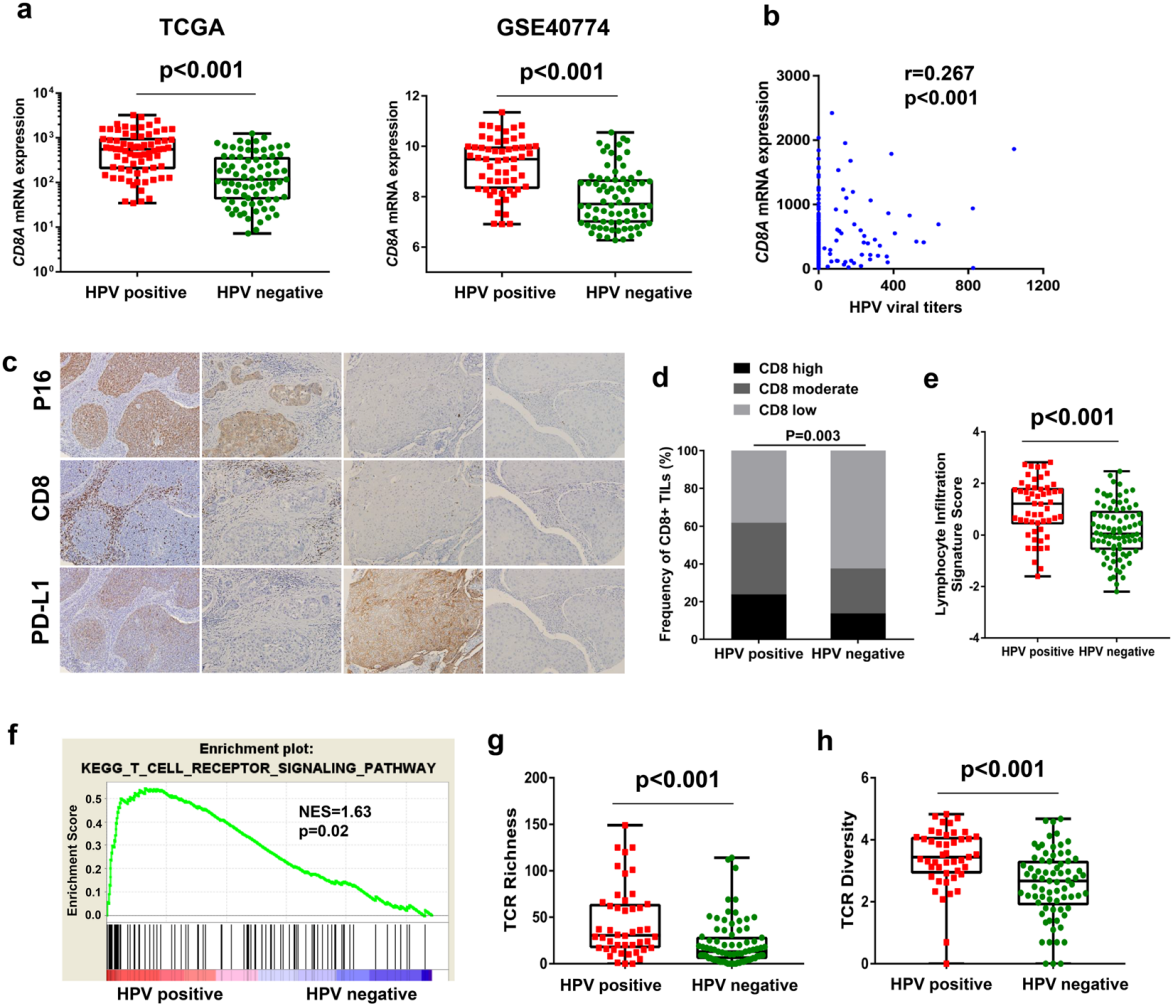
HPV infection promoted T-cell infiltration and T-cell receptor (TCR) diversity. (**a**) Quantitative analysis of *CD8A* RNA expression from RNA-seq profiles in patients with HPV-positive and -negative head and neck squamous cell carcinoma (HNSCC) based on the TCGA and GSE40774 cohorts. (**b**) Correlation analysis of HPV viral titers and *CD8A* expression based on the TCGA cohort. (**c**) Representative images of immunostaining of PD-L1, p16, and CD8 in serial sections of HNSCC tumors. (**d**) Immunohistochemical (IHC) analysis of CD8 protein expression comparing HPV-positive with HPV-negative status in a cohort of 130 resected HNSCC patients. (**e**) Quantitative analysis of lymphocyte infiltration signature score in patients with HPV-positive and -negative HNSCC from the TCGA database. (**f**) Gene set enrichment analysis (GSEA) revealed upregulation of the T-cell receptor signaling pathway in the HPV-positive group compared with the HPV-negative group. (**g**,**h**) Quantitative analysis of TCR diversity and TCR richness in patients with HPV-positive and -negative HNSCC from the TCGA database. TCGA, The Cancer Genome Atlas; NES, normalized enrichment score.

As tumor-infiltrating lymphocytes (TILs) are proven to be a stable and ideal biomarker for response to PD-1 blockade immunotherapy, we evaluated the *CD8A* RNA levels in the HPV-positive and -negative groups based on gene profiles from the TCGA and GSE40774 cohorts. Our analysis confirmed that HPV-positive patients had higher levels of *CD8A* than HPV-negative patients in both cohorts (TCGA and GSE40774: *p* < 0.001; Fig. 4a**)**. Notably, we also detected a positive correlation between HPV viral titers and *CD8A* RNA levels (r = 0.267, *p* < 0.001; Fig. 4b). IHC analysis of CD8^+^ TILs in the 130 HNSCC specimens corroborated the finding that HPV-positive HNSCC patients exhibit greater T-cell infiltration than HPV-negative patients (*p* = 0.003; Fig. 4c,d). To further confirm the relationship between HPV status and T-cell infiltration, we analyzed the lymphocyte infiltration signature score based on data previously published on TCGA^19^. A significant increase was observed in HPV-positive HNSCC compared with HPV-negative (Fig. 4e, *p* < 0.001). We also created a heat map of the relative RNA expression of immunocyte-related core-enriched genes based on the TCGA and GSE40774 cohorts to identify which kind of immune cells was the predominant subset in HPV-positive tumors. We found that HPV-positive status corresponded strongly with the levels of biomarkers of immune effector cells (effector T (T-eff) cells, natural killer (NK) cells, and B cells), but not with those of immune suppressor cells (regulatory T (T-reg) cell and macrophages) (Supplementary Fig. 1A,B). Meanwhile, the analysis of *CD8B*, *CD4* and *IL12* in both TCGA and GSE40774 cohorts furtherly supported that it is CD8+ effector T and NK cells but not CD4+T cell that function as the main part in HPV positive HNSCC (Supplementary Fig. 2).

To determine the underlying mechanism accounting for the response to anti-PD-1/PD-L1 therapy in HPV-associated HNSCC patients, we carried out gene set enrichment analysis (GSEA) to identify pathways enriched in specific HPV status. The T-cell receptor (TCR) pathway that functions in immunological recognition was significantly up-regulated in HPV-positive tumors **(**Fig. 4f, *p* = 0.02**)**. Furthermore, we analyzed the TCR diversity and richness between HPV-positive and -negative tumors based on previously published data^19^. This revealed that HPV-positive HNSCC had significantly increased TCR diversity (Fig. 4g, *p* < 0.001) and richness (Fig. 4h, *p* < 0.001) compared with HPV-negative HNSCC. However, there was no difference in B-cell receptor (BCR) diversity (*p* = 0.087) or richness (*p* = 0.477) between the two groups (Supplementary Fig. 3A,B). These results indicated that HPV infection in HNSCC potentially facilitates T-cell infiltration and immune recognition.

### HPV-positive status correlated with increased cytotoxicity and a T cell-inflamed microenvironment

**Figure 5:**
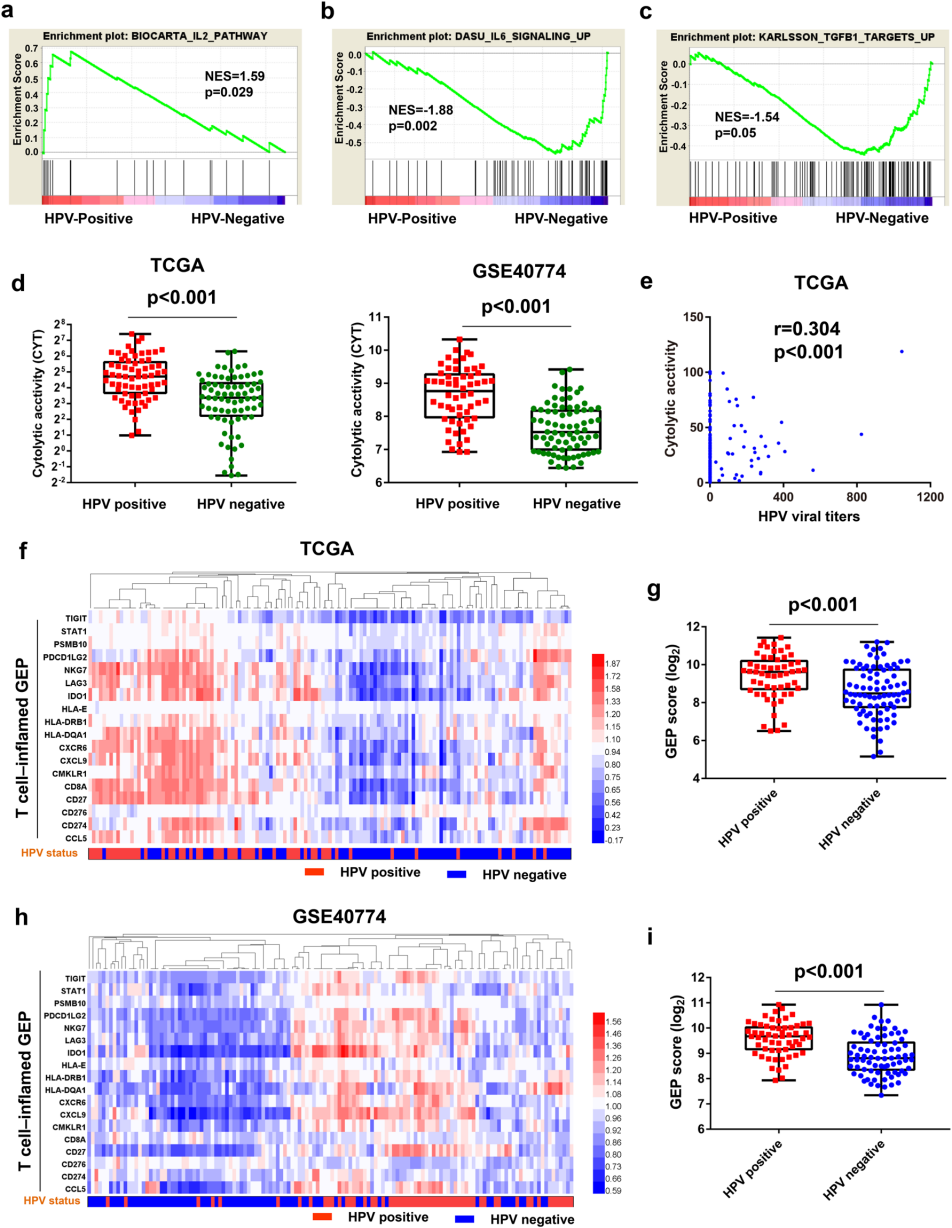
HPV-positive status correlated with increased cytotoxicity and T cell-inflamed gene expression profiles (GEPs). (**a**–**c**) Gene set enrichment analysis (GSEA) revealed upregulation of the IL2 signaling pathway and downregulation of the IL6 and TGFB1 pathways in the HPV-positive group compared with the HPV-negative group. (**d**) Quantitative analysis of cytolytic activity (CYT) in patients with HPV-positive and -negative head and neck squamous cell carcinoma (HNSCC) based on the TCGA and GSE40774 cohorts. (**e**) Correlation analysis of HPV viral titers and CYT based on the TCGA cohort. (**f**,**h**) Heatmap depicting 18 genes representative of T cell-inflamed GEP correlated to the HPV status of the corresponding HNSCC tissues based on the TCGA and GSE40774 cohorts. (**g**,**i**) Quantitative analysis of GEP scores in patients with HPV-positive and -negative HNSCC based on the TCGA and GSE40774 cohorts. TCGA, The Cancer Genome Atlas; NES, normalized enrichment score.

A recent study demonstrated that patients with preexisting immunity—defined by a high interferon gamma (IFN-γ)-associated cytolytic immune signature—had improved OS when treated with a PD-L1 inhibitor^27^. GSEA revealed that the IL-2 pathway, which directly activates the immune response, was significantly upregulated in HPV-positive tumors, whereas the IL-6 and TGFB1 pathways, which negatively regulate the immune microenvironment, were significantly downregulated in HPV-positive tumors (Fig. 5a–c). Furthermore, the data from the TCGA and GSE40774 cohorts proved that an HPV-positive status was associated with higher cytolytic activity (CYT) compared with HPV-negative patients (*p* < 0.001; Fig. 5d). There was also a positive correlation between HPV viral titers and the CYT of HNSCC patients (r = 0.304, *p* < 0.001, Fig. 5e). Thus, we created a heat map of the relative RNA expression levels of inflammatory cytokines between HPV-positive and -negative patients. The IFN-γ-related gene signatures were predominantly concentrated in the HPV-positive areas, whereas the IL6/TGF-β-related gene signatures mostly appeared in the HPV-negative areas. **(**Supplementary Fig. 4A,B).

To obtain a more comprehensive understanding of the relationship between HPV status and the immune microenvironment, we analyzed the T cell-inflamed gene expression profiles (GEPs) that contained 18 genes relating to antigen presentation, chemokine expression, cytotoxic activity and adaptive immune resistance, which were shown to predict response to anti-PD-1–directed therapy^18,28^. A heat map of the relative RNA expression levels of the T cell-inflamed GEP showed increased expression in HPV-positive tumors from the TCGA and GSE40774 cohorts (Fig. 5f,h). Further statistical analysis of GEP score and HPV status supported that HPV-positive status was associated with higher GEP score than HPV-negative status in both the TCGA (Fig. 5g, *p* < 0.001) and GSE40774 (Fig. 5i, *p* < 0.001) databases. These findings suggest that HPV-positive HNSCC patients tended to have a T cell-inflamed microenvironment which contributes to PD-1 blockade immunotherapy sensitivity.

Given that HPV status was significantly correlated with immune-related genes expression. We wonder whether HPV functions as a prognostic agent in HNSCC. A univariate and multivariable cox regression analysis of overall survival in TCGA cohort based on HPV status and key immune-related genes was performed. The results showed HPV status was not an independent prognostic factor for HNSCC survival while some other immune-related genes, such as *CD8A*, *CD4*, *TGFB1* and *CTLA4*, were the independent prognostic factors (Supplementary Table S4). These findings indicated T-cell infiltration or T cell-inflamed microenvironment in HPV positive HNSCC contributed to the efficacy of anti-PD-1 therapy, whereas HPV-infection itself may not alter the survival of HNSCC patients.

## Discussion

Considering the significant toxicity and limited clinical efficacy of traditional platinum-based chemotherapy, novel therapeutic strategies that can broaden the current treatment regime of HNSCC are urgently needed. The development of ICIs has provided a novel treatment option that could also be useful for recurrent patients. Clinical trials have already proven that anti-PD-1/PD-L1 therapy provides antitumor activity and has an acceptable safety profile when used to treat HNSCC patients, although several studies have reported that HNSCC sufferers respond to PD-1/PD-L1 therapy regardless of their HPV status^11^. This study may be the first to confirm the effect of HPV status on anti-PD-1/PD-L1 therapy based on a pooled analysis of prospective clinical trials. More importantly, we performed an integrated analysis based on large data platforms (TCGA, etc.) and clinical samples to clarify the potential mechanisms that account for the HPV-related immunotherapy response, which supported our conclusions.

It has been proposed that HPV-positive HNSCC have a better prognosis than those that are HPV-negative^1,29^. Recent studies have demonstrated that the improved prognosis of HPV-positive HNSCC patients is related to the high responsiveness of these tumors to chemotherapy, radiation and target therapy^7,30,31^. These studies also showed better prognosis in HPV-positive HNSCC patients at every line of therapy, which supported our hypothesis that HPV status may serve as both a prognostic and predictive biomarker in HNSCC. In our study, 4 trials were included in the OS analysis, of which all patients were at second line therapy or above. Our results showed prolonged OS in patients with HPV-positive HNSCC. The analysis of ORR also suggested that HPV-positive HNSCC showed higher response rates than HPV-negative HNSCC, supporting the favorable OS. These results indicate that HPV status may function as both a prognostic and predictive biomarker for ICI treatment and promote an improved prognosis of HPV-positive HNSCC.

The expression of PD-L1 is a useful predictive biomarker for checkpoint inhibitors according to clinical trials^21,32,33^, and higher response rates have been observed in HNSCC patients with high levels of PD-L1 expression^4,11^. In this study, we demonstrated that as a predictive biomarker, HPV is independent of PD-L1 expression. A recent clinical trial on the treatment of recurrent HNSCC with nivolumab revealed that a single-positive PD-L1 or p16 result could predict a better response to nivolumab, whereas a double-positive result could not, which partly corroborates our results^4^. We speculated whether it was possible to determine if HPV status can serve as a specific biomarker to predict the response to ICIs in PD-L1-negative HNSCC patients. In addition, our study demonstrated that HPV infection promoted T-cell infiltration and produced an inflamed microenvironment, which may subsequently induce PD-L1 expression. The dynamic changes of PD-L1 and TIL in HPV-positive tumors potentially overcome the immune ignorance in those with PD-L1-negative tumors. These mechanisms are the most likely explanation for patients with low or no PD-L1 expression that respond well to anti-PD1/PD-L1 therapy.

Previous studies have reported that the HPV-16 E7 oncoprotein was found to induce centrosomal abnormalities, thereby disrupting mitotic fidelity and increasing the risk of chromosome missegregation and aneuploidy^24^. It is well known that chromosomal instability is correlated with an increase in gene mutations. Furthermore, foreign viral antigens from specific viruses were also found to enhance immunogenicity^34^. To investigate HPV-induced responses to anti-PD-1/PD-L1 immunotherapy, we analyzed the relationships between HPV status and TMB, tumor neoantigens and CNA in HNSCC databases, but found no correlations. Possible explanations may be that other agents predominantly affect the TMB in HPV-positive HNSCC, or that only some specific HPV oncoproteins (such as HPV E7) can increase the TMB or alter tumor aneuploidy. These findings suggest that HPV-induced responses to anti-PD-1/PD-L1 therapy may be independent of tumor genomic alternations.

It has been suggested that virus-infected cancer cells communicate with stromal cells through the secretion of cytokines and chemokines, or by releasing tumor exosomes to alter the tumor microenvironment^35,36^. Previous studies have identified the relationship between HPV infection and T-cell-inflamed phenotype^37^. These findings also supported the improved prognosis of patients with HPV-positive HNSCC that probably resulted from activated immune cell subtypes. Our study also verified that HPV-positive status was significantly correlated with lymphocyte infiltration (T-eff cells, NK cells, B cells) and cytolytic activity based on an integrated analysis. However, we also discovered that tumors with HPV infection showed up-regulation of the TCR pathway as well as increased TCR diversity and richness, suggesting enhanced immune recognition. Meanwhile, we observed that HPV positive status was correlated with an increased T cell-inflamed GEP, which was demonstrated to predict the response to anti-PD-1 therapy. Therefore, our study extended the understanding of the effect of HPV status in the immune microenvironment and furtherly verified these pre-clinical perspectives through the analysis of anti-PD-1 therapy for HNSCC trial patients.

Our study has several limitations. Firstly, although we established the relationships between HPV status and PD-L1 expression, tumor mutation profiles and the prevalence of inflammation-related immune cells and molecules, there is a lack of *in vivo-* and *in vitro*-based experimental information to determine the molecular mechanisms underlying HPV-induced immune activation. Our next study will focus on determining the biological mechanisms underlying the relationship between viral infection (HPV, EBV, etc.) and immune surveillance in HNSCC. Secondly, we demonstrated that the HPV-induced immunotherapy response was independent of PD-L1 expression in HNSCC patients. However, we failed to conduct a stratified analysis of the efficacy of HPV status for predicting anti-PD-1/PD-L1 treatment in a subgroup of PD-L1-positive and -negative patients. This limitation arose because the primary data extracted from published studies lacked individual results for HPV and PD-L1 status. Further studies should therefore focus on the value of HPV status for predicting the response to anti-PD-1/PD-L1 therapy in HPV-negative patients or patients with a low TMB.

In conclusion, our study proved that anti-PD-1/PD-L1 treatment is more effective in HPV-associated HNSCC patients. HPV status may be an independent predictive factor in addition to PD-L1 expression and TMB in HNSCC, which activates the immune microenvironment by recruiting infiltrated T-cells and promoting their CYT.

## Supplementary information

supplementary Dataset

## Data Availability

The datasets generated during and/or analyzed during the current study are available from the corresponding author on reasonable request.
