# Supplementary material for: HPV-positive status associated with inflamed immune microenvironment and improved response to anti-PD-1 therapy in head and neck squamous cell carcinoma
Source: Sci Rep. 2019 Sep 16;9:13404. doi: 10.1038/s41598-019-49771-0 (PMC6746709; doi:10.1038/s41598-019-49771-0)
Supplement: Supplementary file 1 — supplementary Dataset [file 41598_2019_49771_MOESM1_ESM.pdf]

HPV-positive status associated with inflamed immune microenvironment and improved response to anti-PD-1 therapy in head and neck squamous cell carcinoma

Jian Wang [1, *], Hao Sun[2, *], Qin Zeng [1], Xue-Jun Guo[1], Hui Wang[1], Huan-Huan Liu[1], Zhong-Yi Dong[1]

Supplementary Fig. 1

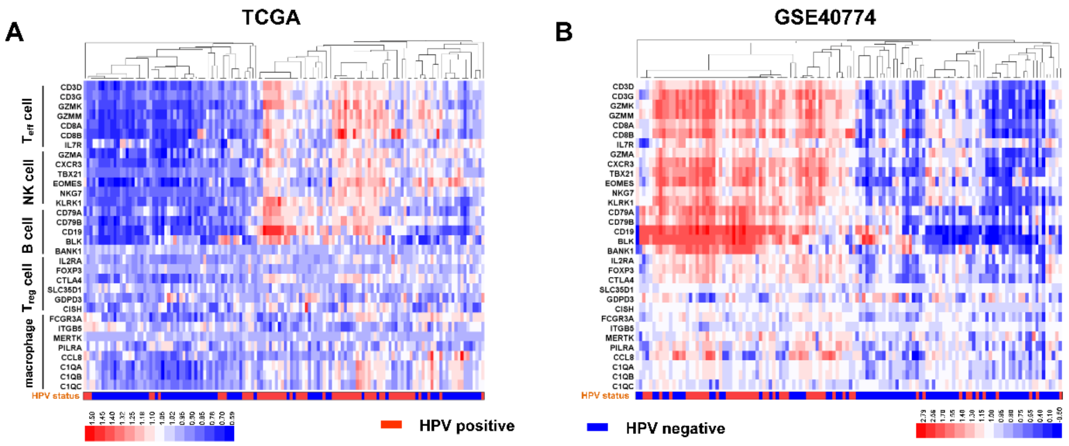

Figure S1. Heatmap depicting RNA expression of specific genes related to effector T-cells, natural killer (NK) cells, B cells, regulatory T-cells, and macrophages, and HPV status in corresponding HNSCC tissues based on the TCGA (a) and GSE40774 (b) cohorts. T_{eff}, effector T; T_{reg}, regulatory T; TCGA, The Cancer Genome Atlas.

Supplementary Fig. 2

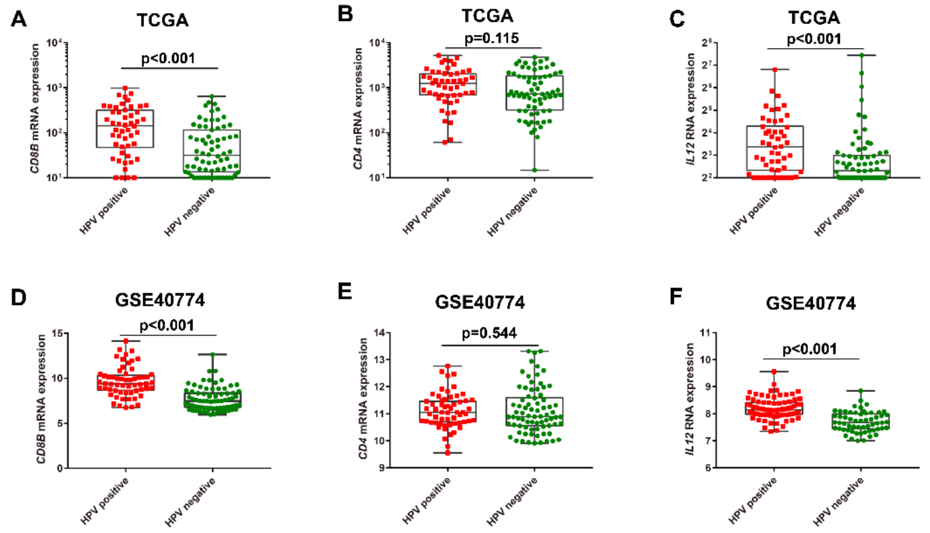

Figure S2. Quantitative analysis of (a and d) CD8B, (b and e) CD4, and (c and f) IL12 RNA expression from RNA-seq profiles in patients with HPV-positive and -negative HNSCC based on The Cancer Genome Atlas (TCGA) and GSE40774 database.

Supplementary Fig. 3

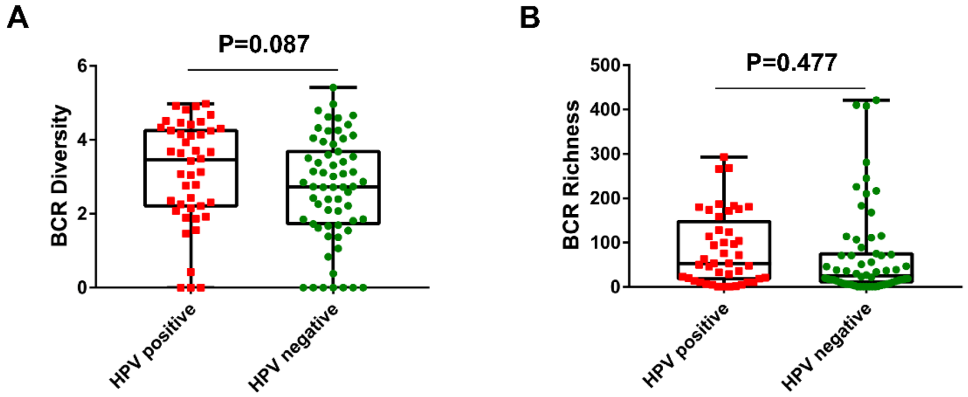

Figure S3. Quantitative analysis of B-cell receptor (BCR) diversity (a) and BCR richness (b) in patients with HPV-positive and -negative HNSCC based on the TCGA database.

Supplementary Fig. 4

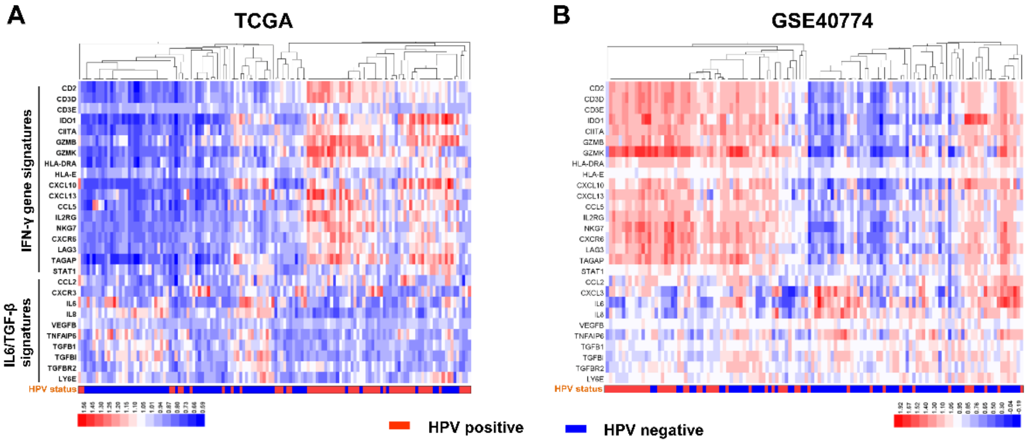

Figure S4. Heatmap depicting specific gene signatures related to IFN-γ, IL6, and TGF-β pathways, and HPV status in the corresponding HNSCC tissues based on the TCGA (a) and GSE40774 (b) cohorts.

Supplementary Table 1. Additional publicly available data sets used in the study

Cohorts	Samples; HPV	Source	Reference
TCGA	451<br>HPV+(77);<br>HPV-(78)<br>Unknown (296)	TCGA database portal	Cell. 2015 Jan 15;160(1-2):48-61.
GSE40774	134<br>HPV+(58);<br>HPV-(76)	GSE40774<br>GPL13497	Clin Cancer Res 2015 Feb 15;21(4):870-81.
MSK-IMPACT	52<br>HPV+(21);<br>HPV-(31)	cBioPortal	JAMA Oncol. 2017;3(2):244-255.
GSE62027	7 HNSCC cells<br>HPV+(3);<br>HPV-(4)	GSE62027<br>GPL570	Oncotarget 2016 Nov 8;7(45):73845-73864.

Supplementary Table 2. Demographic and clinicopathologic characteristics of head and neck squamous cell carcinoma in 4 independent cohorts.

	TCGA	GSE40774	MSK-IMPACT	HNSCC-TMA
**No. of patients**	451	134	53	130
**Age**				
**Mean, yrs**	61.0	57.0	59.0	62.0
**Range**	19 – 90	34 – 80	16 – 99	34 – 90
**Gender**				
**Male**	328 (72.7)	107 (79.9)	39 (73.6)	85 (65.4)
**Female**	123 (27.3)	23 (17.2)	14 (26.4)	45 (34.6)
**Smoking**				
**Light/Never**	98 (21.7)	58 (43.3)	24 (45.3)	
**Heavy**	341 (75.6)	72 (53.7)	29 (54.7)	
**Unknown**	12 (2.7)	4 (3)		

**Stage**				
**I-II**	105 (23.3)	2 (1.5)	8 (15.1)	28 (21.5)
**III**	99 (21.9)	3 (2.2)	4 (7.5)	34 (26.2)
**IV**	234 (51.9)	124 (92.6)	37 (69.8)	56 (43.1)
**Unknown**	13 (2.9)	5 (3.7)	4 (7.6)	12 (9.2)
**Primary sites**				
**Oral Cavity**	267 (59.2)	25 (18.6)	20 (37.7)	84 (64.6)
**Oropharynx**	76 (16.8)	75 (56.0)	18 (34.0)	10 (7.7)
**Hypopharynx**	9 (2.0)	1 (0.7)	2 (3.8)	0
**Larynx**	99 (22.0)	29 (21.7)	7 (13.2)	30 (23.1)
**Other**	0	4 (3.0)	6 (11.3)	6 (4.6)
**HPV status**				
**Positive**	77 (17.1)	55 (41.0)	21 (39.6)	21 (16.2)
**Negative**	78 (17.3)	75 (56.0)	31 (58.5)	109 (83.8)
**Unknow**	296 (65.6)	4 (3.0)	1 (1.9)	0

Abbreviations: TCGA, The Cancer Genome Atlas; HNSCC-TMA, head and neck squamous cell carcinoma-tissue microarray; MSK-IMPACT, Memorial Sloan Kettering-Integrated Mutation Profiling of Actionable Cancer Targets; NA, not available.

Supplementary Table 3. Characteristics of trials included in the pooled-analyses.

Clinical trial	Phase	Treatment	Primary sites	HPV status	Median OS (months)	ORR (%)
CheckMate-141[1]	III	Nivolumab (n=240)	Larynx (34)<br>Oral cavity (108)<br>Pharynx (92)<br>Other (6)	HPV+ (64)<br>HPV- (56)	9.1<br>7.5	17.2%<br>14.3%
KEYNOTE-012[2]	Ib	Pembrolizumab (n=60)	Oropharynx (16)<br>Tongue (14)<br>Oral cavity (11)<br>Other (19)	HPV+ (23)<br>HPV- (37)	not reached<br>8	25%<br>19%
KEYNOTE-012 Expansion[3]	Ib	Pembrolizumab (n=132)	Oropharynx (60)<br>Oral cavity (17)<br>Larynx (16)<br>Hypopharynx	HPV+ (28)<br>HPV- (104)		32%<br>14%

			(12)<br>Other (25)			
KEYNOTE-055[4]	II	Pembrolizumab (n=171)	Hypopharynx (7)<br>Larynx (30)<br>Oral cavity (28)<br>Oropharynx (100)<br>Other (6)	HPV+ (37)<br>HPV- (131)	11<br>6	16%<br>15%
NCT01693562[5]	Ia	Atezolizumab (n=32)	Oral cavity (7)<br>Oropharynx (18)<br>Other (7)	HPV+ (13)<br>HPV- (12)		15%<br>17%
NCT01375842[6]	II	Durvalumab (n=112)	Oropharynx (40)<br>Oral cavity (47)<br>Larynx (15)<br>Hypopharynx (9)<br>Other (1)	HPV+ (34)<br>HPV- (65)	10.2<br>5	29.4%<br>10.8%

Supplementary Table 4. Univariate and multivariable cox regression analysis of HPV status and immune-related genes expression for overall survival in TCGA cohort with head and neck squamous cell carcinoma.

	**Univariate analysis**			**Multivariate analysis**		
**Variable**	**HR**	**95%CI**	***P*-value**	**HR**	**95%CI**	***P*-value**
HPV (Positive *vs.* Negative)	0.48	0.23-1.03	0.060			
PD-L1 (High *vs.* low)	0.58	0.29-1.15	0.119			
CD8A (High *vs.* low)	0.31	0.15-0.67	0.003	0.26	0.09-0.80	0.019
CD8B (High *vs.* low)	0.36	0.17-0.76	0.007			
CD4 (High *vs.* low)	0.31	0.15-0.67	0.003	0.21	0.06-0.70	0.011
IFNG (High *vs.* low)	0.41	0.20-0.83	0.014			

CTLA4 (High *vs*. low)	0.55	0.28-1.10	0.091	5.54	1.67-18.45	0.005
TGFB1(High *vs*. low)	0.53	0.27-1.05	0.069	0.43	0.21-0.89	0.024
FOXP3 (High *vs*. low)	0.50	0.25-0.99	0.048			
HLA-E (High *vs*. low)	0.59	0.30-1.16	0.127			

Abbreviations: HR, hazard ratio; CI, confidence interval. High *vs*. low, the definition of high and low expression was chosen as median of each gene expression.
